# *In Silico* Pharmacogenomic Assessment of Glucagon-like Peptide-1 (GLP1) Agonists and the Genetic Addiction Risk Score (GARS) Related Pathways: Implications for Suicidal Ideation and Substance Use Disorder

**DOI:** 10.2174/011570159X349579241231080602

**Published:** 2025-01-24

**Authors:** Alireza Sharafshah, Kai-Uwe Lewandrowski, Mark S. Gold, Brian Fuehrlein, John Wesson Ashford, Panayotis K. Thanos, Gene Jack Wang, Colin Hanna, Jean Lud Cadet, Eliot L. Gardner, Jag H. Khalsa, Eric R. Braverman, David Baron, Igor Elman, Catherine A. Dennen, Abdalla Bowirrat, Albert Pinhasov, Edward J. Modestino, Paul R. Carney, Rene Cortese, Rossano Kepler Alvim Fiorelli, Sergio Schmidt, Aryeh R. Pollack, Rajendra D. Badgaiyan, Kenneth Blum

**Affiliations:** 1 Cellular and Molecular Research Center, School of Medicine, Guilan University of Medical Sciences, Rasht, Iran;; 2 Department of Orthopaedics, Fundación Universitaria Sanitas Bogotá D.C. Colombia;; 3 Division Personalized Pain Research and Education, Center for Advanced Spine Care of Southern Arizona, Tucson, AZ., 85712, USA;; 4 Department of Psychiatry, Washington University School of Medicine, St. Louis, MO., 63110, USA;; 5 Department of Psychiatry, Yale University School of Medicine, New Haven CT., 06511, USA;; 6 Department of Psychiatry & Behavioral Sciences, Stanford University, Palo Alto, CA Director, War Related Illness & Injury Study Center, VA Palo Alto Health Care System, Palo Alto, CA, 94305, USA;; 7 Behavioral Neuropharmacology and Neuroimaging Laboratory on Addictions, Clinical Research Institute on Addictions, Department of Pharmacology and Toxicology, Jacobs School of Medicine and Biosciences, State University of New York at Buffalo, Buffalo, NY., 14260, USA;; 8 Laboratory of Neuroimaging, National Institute of Alcohol Abuse & Alcoholism, Bethesda, MD, 20892, United States;; 9 Molecular Neuropsychiatry Research Branch, NIH National Institute on Drug Abuse, Bethesda, MD., 20892, USA;; 10 Neuropsychopharmacology Section, Intramural Research Program, National Institute on Drug Abuse, National Institutes of Health, Baltimore, MD., 20892, USA;; 11 Division of Therapeutics and Medical Consequences, Medical Consequences of Drug Abuse and Infections Branch, National Institute on Drug Abuse, NIH, Special Volunteer, Industrial Drive, Gaithersburg, MD., 20892, The USA;; 12 Department of Microbiology, Immunology, and Tropical Medicine, The George Washington University School of Medicine, Washington, NWDC., 20037, The USA;; 13 The Kenneth Blum Behavioral & Neurogenetic Institute, LLC., Austin, TX., 78701, USA;; 14 Center for Sports, Exercise, Mental Health, Western University Health Sciences, Lebanon, OR., 91766, USA;; 15 Cambridge Health Alliance, Harvard Medical School, Cambridge, MA., 02115, USA;; 16 Department of Family Medicine, Jefferson Health Northeast, Philadelphia, PA., USA;; 17 Department of Molecular Biology, Adelson School of Medicine, Ariel University, Ariel, Israel;; 18 Brain & Behavior Laboratory, Department of Psychology, Curry College, Milton, MA., 02186, USA;; 19 Departments of Pediatrics and Neurology, University of Missouri School of Medicine, Columbia, 65212, Missouri, USA;; 20 Departments of Pediatrics and Obstetrics, Gynecology and Women's Health. School of Medicine. University of Missouri, Columbia, MO, 65212, USA;; 21 Department of General and Specialized Surgery, Gaffrée e Guinle University Hospital, Federal University of the State of Rio de Janeiro (UNIRIO), Rio de Janeiro, Brazil;; 22 Post-Graduate Program in Neurology, Federal University of the State of Rio de Janeiro, Rio de Janeiro, Brazil;; 23 Department of Psychiatry, Texas Tech University Health Sciences, School of Medicine, Midland, TX., 79430, USA;; 24 Department of Psychiatry, Wright State University Boonshoft School of Medicine and Dayton VA Medical Center, Dayton, OH., 45435, USA;; 25 Department of Psychiatry, Human Integrated Services Unit, University of Vermont Center for Clinical & Translational Science, College of Medicine, Burlington, VT, 05401, USA

**Keywords:** GLP1, Ozempic, suicide ideation, substance use disorder, dopamine homeostasis, in-depth *in silico*

## Abstract

**Introduction:**

Glucagon-Like Peptide-1 Receptor (GLP1R) agonists have become widespread anti-obesity/diabetes pharmaceuticals in the United States.

**Aim:**

This article aimed to provide our current knowledge on the plausible mechanisms linked to the role of Ozempic (Semaglutide), which is generalized as one of the anti-addiction compounds.

**Methods:**

The effects of GLP1R agonists in Alcohol Use Disorder (AUD) and substance use disorder (SUD) are mediated, in part, through the downregulation of dopamine signaling. We posit that while GLP1R agonism could offer therapeutic advantages in hyperdopaminergia, it may be detrimental in patients with hypodopaminergia, potentially leading to long-term induction of Suicidal Ideation (SI). The alleged posit of GLP1 agonists to induce dopamine homeostasis is incorrect. This study refined 31 genes based on the targets of Ozempic, *GLP1R*, and related enzymes for SI and 10 genes of the Genetic Addiction Risk Score (GARS) test. STRING-MODEL refined 29 genes, and further primary analyses indicated associations of *GLP1R* with *DRD3*, *BDNF*, *CREB1*, *CRH, IL6*, and *DPP4*.

**Results:**

In-depth silico enrichment analysis revealed an association between candidate genes and depressive phenotypes linked with dopaminergic signaling. Finally, through primary and in-depth silico analyses, we demonstrated multiple findings supporting that GLP1R agonists can induce depression phenotypes.

**Conclusion:**

Our findings suggest that associated polymorphisms seem to have overlapping effects with addictive behaviors of Reward Deficiency Syndrome (RDS) and dopamine regulation. Consequently, GLP1R agonists may represent a double-edged sword, potentially triggering both anti-addictive effects and SI by exacerbating depressive phenotypes. Thus, we encourage the scientific community to perform further empirical clinical studies to confirm this proposed pathway.

## 1 INTRODUCTION

Glucagon-like peptide-1 Receptor (GLP1R) agonists have become extensive anti-obesity/diabetes medications in the United States. This study aimed to investigate the potential mechanisms associated with Ozempic (Semaglutide), which is generalized as one of the anti-addiction compounds. The impacts of GLP1R agonists on substance use disorder (SUD) and alcohol use disorder (AUD) are partially mediated by dopamine signaling downregulation. We hypothesize that, whereas GLP1R agonism may be beneficial for treating hyperdopaminergia, it may be detrimental for treating hypodopaminergia, perhaps resulting in the long-term induction of suicidal ideation (SI). Thus, by examining prior reports and our analyses, we will investigate the aforementioned idea.

**Fig. (1):**
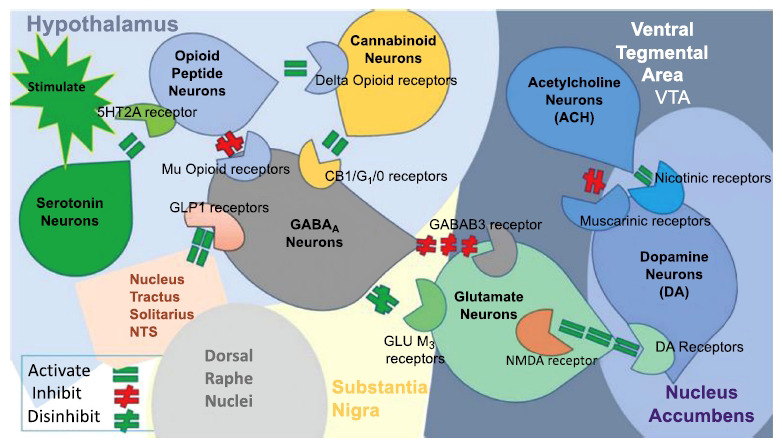
The mesolimbic brain reward cascade. This figure illustrates the interaction of some well-known brain reward cascade (BRC) neurotransmitter pathways. Environmental stimulation initiates the release of serotonin (5-HT) in the hypothalamus, which in turn, for example, *via* 5 HT-2 A receptors activates (green equal sign) the subsequent release of opioid peptides from opioid peptide neurons. Then, in the Substantia Nigra (SN), the opioid peptides bind to two distinct opioid receptors with different mechanism of action. One is through the mu-opioid receptor that inhibits (red hash sign) GABA-ergic neurons (possibly *via* an opioid peptide like enkephalins). The second stimulates cannabinoid neurons (for example, the Anandamide and 2-arachidonoylglycerol) (green equal sign) through beta-endorphin-linked delta receptors, which inhibit GABAA neurons. When activated, cannabinoids, primarily the 2-arachidonoylglycerol neurons, can disinhibit (green hash sign) GABA-ergic neurons indirectly by CB1 receptor activation. Glutamatergic neurons in the Dorsal Raphe Nuclei (DRN) disinhibit GABA neurons in the SN indirectly through GLU M3 receptor activation (green hash sign). When disinhibited, GABAA neurons will powerfully inhibit (red hash signs) VTA glutaminergic drive *via* GABAB 3 receptors. At the Nucleus Accumbens (NAc), Acetylcholine (ACH) neurons may inhibit (red hash sign) muscarinic and stimulate Nicotinic (green hash) receptors. Glutamate neurons in the VTA will project to dopamine neurons through NMDA receptors (green equal sign) to release dopamine at the NAc. (with permission from Blum *et al*.).

Globally, it is estimated that while 1 in 8 people globally are considered obese, close to 300 million individuals suffer from AUD, including approximately 150 million individuals with alcohol dependence [1, 2]. Most compelling is that the World Health Organization (WHO) estimated that the harmful qualities of alcohol use and abuse account for approximately 5% of premature fatalities. As such, the WHO notes that alcohol use and abuse is a leading cause of preventable fatalities worldwide. It is considered to be linked to consumers of alcohol having a lower rate of treatment than other psychiatric disorders [3]. It is also true that AUD is linked to a high premature mortality rate, which has been associated with dopamine dysregulation, including the *DRD2 A1* allele, which is over-represented in alcohol-dependent individuals [4]. In one study, this allele has also been associated with a highly increased mortality rate in alcohol-dependent individuals [4]. Along similar lines, it has been estimated by the United Nations 2023 World Drug Report that globally, over 354 million humans will use illicit drugs in 2021 [5]. Out of the 354 million, 218 million were attributed to cannabis, 60 million to opioids, 21 million to cocaine, 20 million to ecstasy, and 35 million to amphetamine [5]. These statistics are morphed by the unfortunate fact that the prevalence of tobacco use is over 1 billion. Tobacco use is one of the highest preventable causes of premature death, while eight million humans die due to tobacco‐related diseases annually [6-8]. While there are FDA pharmaceuticals approved for alcohol. Opioid, and tobacco misuse, albeit these treatments are not without adverse effects and even addiction liability (*e.g*., methadone). There is currently no treatment for cannabis or psychostimulant disorders. The search for safe and effective modalities to treat all Reward Deficiency Syndrome (RDS) behaviors continues to be actively explored. Research has shown that people suffering from substance and non-substance behavioral addictions (*i.e*., obesity/food addiction) have overlapping brain dysregulation and share genetic and epigenetic mechanisms, particularly hypodopaminergia [9, 10]. Therefore, hypodopaminergia could exist in an astonishing number of people not only in the USA but also globally. While Howes & Kapur [10] correctly point out that dopamine is currently the only known finite reward molecule, its regulation, and neuronal release are a function of many risk factors beyond DNA antecedents that are linked to reward deficiency [9]. These include but are not limited to stress, trauma, obstetric complications, drug use, genetic and epigenetic insults that reduce presynaptic striatal dopaminergic function. Our proposal is to balance brain neurotransmitters by using positive modulation targeted at both upstream and downstream pathways in order to overcome substance and non-substance behavioral addictions. Thus providing normal or close to normal dopamine release at the various brain regions, especially the Ventral Tegmental Area (VTA) and Nucleus Accumbens (NAc). In terms of the Brain Reward Cascade (BRC) that was first published in 1989, it is noteworthy to add glucagon-like peptide 1 (GLP-1) receptors to the model. One advantage of the GLP-1 receptor agonists in relation to obesity is that they play a significant role in inducing insulin secretion after meals and aid in weight loss by encouraging satiety and delaying stomach emptying [11-13]. In addition, research has shown that GLP-1 receptor agonists are very effective at reducing A1c and cardiovascular disease in patients with existing Atherosclerotic Cardiovascular Disease (ASCVD) [14-21]. Research has also shown that GLP-1 receptor agonists have an overall decrease in mortality in individuals with cardiovascular disease and type 2 diabetes [22, 23]. Furthermore, many have begun to take a keen interest in the role of GLP-1 receptors and dopaminergic regulation in the mesolimbic system of the human brain [24, 25] and the potential role of GLP-1 in the attenuation of, for example, alcohol intake from both human genetic association studies and mouse models of alcohol dependence. However, while there are some reports about the various agonists of GLP-1 with some positive effects in curtailing food intake [12] and even AUD [26, 27], the agonistic effects of GLP-1 are very complex (Fig. **1**). Interestingly, Glucagon-like peptide 1 (GLP-1) regulates food intake, insulin production, and metabolism. Fortunately, a recent study demonstrated that pancreatic α-cells-secreted (intraislet) GLP-1 effectively promotes maternal insulin secretion and metabolic adaptation during pregnancy. Specifically, Qiao *et al*. [28, 29] showed a progressive reduction in maternal blood GLP-1 concentration. GLP-1 receptor agonist injection in late pregnancy significantly reduced fetal body weight, even after restoration of maternal blood glucose concentration. GLP-1 receptor activation significantly reduced the placental labyrinth area, expression of some nutrient transporters, and capillary development. Qiao *et al*. suggested that reducing maternal blood GLP-1 levels is a physiological adaptation process that benefits placental development and fetal growth [28].

Impairment of glucoregulatory function, which is consequential to the addiction neuropathology, can worsen Substance Use Disorder (SUD) and polydrug abuse. Therefore, it is reasonable to expect amelioration of addictive behaviors/ symptoms through the improvement of metabolic function (*i.e*., using GLP-1 receptor agonists). Research has demonstrated that the effects of GLP-1 in alcohol use disorder and SUD are mediated partly through the downregulation of dopamine signaling and even enhanced catabolism in both the synapse and mitochondria due to GLP1 agonistic increase in the gene expression such as Catechol-O-methyltransferase (COMT) and Monoamine Oxidase A (MAOA) [30, 31]. While this may have some important relevance in the short term, where hyperdopaminergia exists, it is important to caution against promoting chronic stimulation *via* a GLP-1 agonist. Recent findings have shown a link between GLP-1 receptor agonists and several genes within the dopaminergic pathway. Therefore, stimulation of GLP-1 receptor agonists, especially chronically, can lead to the dysregulation of the dopaminergic pathway, which in turn can cause depression, suicidality, and other mood disturbances [9, 16, 32-40].

In sum, while employing GLP-1 agonism may offer therapeutic benefits in instances of recognized hyperdopaminergia, it could be determinantal in patients with hypodopaminergia. Most recently, Klausen *et al*. [9, 41] suggested that the therapeutic effects of GLP1R agonists on AUD and SUD seem to be mediated partly through the downregulation of dopaminergic signaling [41]. While this may have some important relevance in the short term, we would like to point out a cautionary pitfall in promoting chronic stimulation *via* a GLP1R agonist, like Exenatide, as an anti-craving agent.

It behooves the scientific community to understand the complexity of GLP1R’s physiology and molecular neurobiological actions. Importantly, the scientific and clinical community are actively exploring both the neurogenetic and epigenetic mechanisms related to, for example, ‘pre-addiction” polymorphic profiles. Understanding the potential of people born with DNA-linked GARS genes and, hence, hypodopaminergia is crucial. Having this DNA data may provide important genetic information related to both those who require and benefit from GLP1R agonistic therapy. It is our hypothesis that in patients with genotypes displaying low dopamine function *via* reward gene polymorphisms assessed with GARS, the long-term administration of GLP1 agonists should be cautioned. This notion is based on reported experiments indicating GLP1 agonists induce dopamine dysregulation (PUBMED 69 articles listed using word term GLP1 agonists and dopamine) [42] that requires possibly modified dosage to prevent any further downregulation of dopamine release at the nucleus accumbens (Fig. **1**).

Accordingly, Klausen *et al*. [34] showed that exenatide significantly attenuated fMRI alcohol cue reactivity in the ventral striatum and septal area, which are critical brain regions for drug reward and addiction, even though it did not significantly reduce the number of heavy drinking days when compared with placebo in AUD patients who underwent functional MRI (fMRI) and single-photon emission CT (SPECT) brain scans. This work further suggested the induction of a blunted reward system following the action of GLP1agonists. Numerous studies have unfortunately shown that blunted brain reward circuitry in the mesolimbic system increases a person's susceptibility to addiction [35-41].

Along these lines, it is indeed noteworthy that in at least 1,580 articles listed in PUBMED using the term “Reward Deficiency” (by the date: 28 June 2024), the primary culprit is a reduced dopaminergic function (hyperdopaminergic), that leads to aberrant craving behavior for both substance and non-substance behavioral addictions. Studies have shown that the exenatide (exendin-4) that passes the blood-brain barrier binds to GLP1R expressed on the inhibitory GABAergic medium spiny neurons in the NAc shell [11, 43-62].

It is known that GLP-1 analogs act to attenuate food intake and body weight, including various hypothalamic nuclei (arcuate nucleus of the hypothalamus, periventricular hypothalamus, lateral hypothalamic area), hindbrain nuclei (parabrachial nucleus, medial nucleus tractus solitarius), hippocampus (ventral subregion; vHP), and nuclei embedded within the mesolimbic reward circuitry (VTA and NAc) [63]. Accordingly, Volkow *et al*. pointed out that disinhibition/stimulation of dopaminergic VTA neurons plays a role in the reinforcing effects of alcohol, nicotine, and opioid [12]. In fact, it has been observed that GLP1Rs are localized on mouse cortical and hippocampal synaptic boutons, specifically on glutamatergic and GABAergic nerve terminals. Activation by exendin-4 increased the release of either [^3^H]d-aspartate or [^3^H]GABA [14]. It is well-known that in the mesolimbic system, enhancing GABAergic function and neuro-activation significantly reduces NAc dopamine release [15].

Specifically, it was reported that both diazepam and muscimol attenuate dopamine release and that decreases in dopamine release following GABA_A_ receptor activation is inhibited by co-application of a GABA_B_ receptor antagonist. These findings support the notion that activation of GABA_A_ receptors in the NAc reduces dopamine release by disinhibition of local GABA signaling and subsequent activation of GABA_B_ receptors [64].

Research has shown that GLP1R agonists can affect binge-like alcohol drinking. Chuong *et al*. reported that semaglutide dose-dependently reduced binge-like alcohol drinking in mice [16]. A similar effect was also observed on the intake of other caloric/noncaloric solutions. Semaglutide also reduced binge-like and dependence-induced alcohol drinking in rats. Additionally, semaglutide increased sIPSC frequency in CeA and ILC neurons from alcohol-naive rats, suggesting enhanced GABA release. The authors concluded that the GLP1R analogue semaglutide decreased alcohol intake across different drinking models and species and modulated central GABA neurotransmission, which would reduce dopaminergic signaling and could then lead to “psychological extinction” [16].

Finally, as of July 2023, the European Medicines Agency (EMA) has begun a genetic analysis through reviewing data regarding the risk of suicidal thoughts and thoughts of self-harm associated with GLP1R agonists, including Ozempic (semaglutide), Saxenda (liraglutide), and Wegovy (semaglutide) [65]. The Icelandic Medicines Agency prompted this genetic analysis in response to reports of suicidal thoughts and self-harm among patients taking liraglutide and semaglutide medications. Authorities are currently reviewing approximately 150 reports of potential suicide attempts and self-harm incidents. Furthermore, from 2010-2023 the FDA has received 265 reports of suicidal thoughts in patients taking GLP1R agonists, particularly tirzepatide, liraglutide, and semaglutide, prompting a review of these medications [66]. Additionally, a recent retrospective pharmacovigilance study of the European Pharmacovigilance database was conducted for the period from 1 January 2018 to 10 July 2023 [67]. Disproportionality analyses (reporting odds ratio, ROR) were performed to assess the reporting probability of suicidal events among GLP-1 RAs. A total of 230 reports of suicidal events were identified. The most reported GLP-1 RA was liraglutide (38.3%), followed by semaglutide (36.5%) and dulaglutide (16.1%). The most reported events were suicidal ideation (65.3%) and suicide attempt (19.5%). Disproportionality analysis found a higher reporting probability of suicidal events for semaglutide than dulaglutide (ROR, 2.05; 95% CI, 1.40-3.01) and exenatide (ROR, 1.81; 95% CI, 1.08-3.05). Suicidal events were mostly reported with semaglutide and liraglutide, which were also associated with significantly higher reporting probabilities compared to other GLP1 RAs. In another study [68], during the study period, 31,444 adverse event reports were identified: semaglutide (n = 13,956; 44.4%), liraglutide (n = 16,748; 53.2%), and tirzepatide (n = 740; 2.3%). There were 372 reports with psychiatric adverse event reports (n = 372; 1.18%) with a total of 481 adverse events. Women accounted for 65% (n = 242) of these reports. Depression was the most commonly reported adverse event (n = 187; 50.3%), followed by anxiety (n = 144; 38.7%) and suicidal ideation (n = 73; 19.6%). Nine deaths (8 with liraglutide and 1 with semaglutide) and 11 life-threatening outcomes (4 associated with liraglutide and 7 with semaglutide) were reported. The fatal outcomes occurred primarily among men (8 out of 9), resulting from completed suicidal attempts and depression. Furthermore, various pharmacovigilance analyses and notifications from different regulatory agencies have documented the occurrence of suicidal thoughts and self-injurious behavior associated with the use of GLP-1 receptor agonists [69]. Maideen and Rashid proposed that healthcare professionals should be aware of GLP-1 receptor agonists related to suicidal thoughts and self-injurious behavior. Patients should not misuse/abuse antidiabetic GLP-1 receptor agonists and should consult their physician before using any GLP-1 receptor agonists for weight loss [69-76].

In contrast, others could not find an association between GLP1 agonists and SI or suicide attempts [77-80]. Indeed, this is not surprising in terms of our cautionary note concerning attenuation rather than chronic promotion of enhanced dopamine signaling, potentially leading to hypodopaminergia and potential SI. However, while there is reason for concern establishing causality requires further investigation, which will probably be addressed by the Pharmacovigilance Risk Assessment Committee of the European Medicine Agency and the FDA in the future, we performed *in silico* pharmacogenomic assessment of GLP1 agonists and GARS related pathways to potentially uncover glp1 agonistic induced suicidality.

## 2 METHODS

The current paper employed a filtering strategy by including candidate genes from various sources, including DrugBank (https://go.drugbank.com/), GeneCards (https://www.genecards.org/), and our well-described Genetic Addiction Risk Score (GARS) test. The rationale behind these sources involved the basic interaction of a medication as a ligand (*e.g*., Ozempic) and potential targets (*e.g*., receptors); accordingly. The initial idea of preparing the primary gene list was to consider all of the Ligand/Receptor molecular compounds based on the curated databases and reliable sources. DrugBank is a well-known database in pharmacological sciences that focuses on FDA-approved compounds. This database has a Ligand/Receptor nature of data availability.

GeneCards is a strong, validated, widespread database with a genetic focus. Utilizing this databank, we obtained highly associated genes with related phenotypes, such as Suicide Ideation (SI), based on the relevance score. The theory behind the relevance score is as follows: Lucene (and thus Elasticsearch) employs the Boolean model to identify matching documents and a formula called the” practical scoring function” to estimate the relevance. This formula uses concepts from the term” frequency/inverse document frequency and the vector space model. “Still, it adds more modern characteristics such as a coordination factor, field length normalization, and term or query clause boosting.

Finally, the GARS gene panel, directly derived from these measurable genes and associated risk polymorphisms, is known to help identify many psychological/addictive manifestations. Moreover, since its inception in 2014, the GARS 10 gene panel has been shown to predict issues regarding the genetic basis of dopamine homeostasis, Substance Use Disorder (SUD), Alcohol Use Disorder (AUD), addiction, and specifically, major depression which has been previously documented abundant publications (*e.g*., GWAS) (Table **S1**). An initial list containing 35 genes was found:. After duplication filtering, 31 genes remained, and by STRING-MODEL (https://string-db.org/) refined to 29 connected genes. By NetworkAnalyst (https://www.networkanalyst.ca/NetworkAnalyst/), Gene-Regulatory Networks (GRNs) carried out in Gene-microRNA (miRNA) Interactions (GMIs) based on miRTarBase v.8.0. and TF-miRNA coregulatory based on RegNetwork repository as sub-analyses. Deep *in silico*/enrichment analyses (https://maayanlab.cloud/Enrichr/) were performed in three layers: Pathway analysis, Gene Ontology (GO), and Disease/Drug analysis. Pathway analysis utilized Reactome (https://reactome.org/), Kyoto Encyclopedia of Genes and Genomes (KEGG) (https://www.genome.jp/kegg/), and Panther (https://www.pantherdb.org/) databases. GO used GO Biological Process, GO Cellular Component, and GO Molecular Function; Disease/Drug Analysis employed DisGENET (https://www.disgenet.org/home/), GeDiPNet 2023 (http://gedipnet.bicnirrh.res.in/), and Jensen DISEASE (https://diseases.jensenlab.org/Search) databases (Table **1**).

**Fig. (2):**
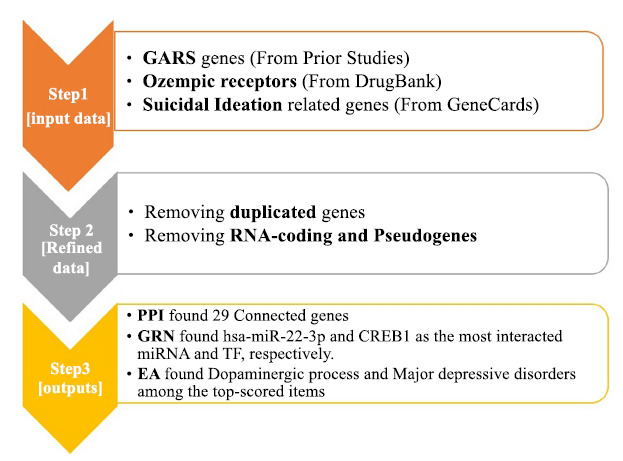
Flowchart of method strategy in a summarized status. **Abbreviations**: GARS: Genetic Addiction Risk Severity; PPI: Protein-Protein Interaction; GNR: Gene Regulatory Networks; and EA: Enrichment Analysis.

Along with Table **1**, we visualized a flowchart representing the process of analysis strategy (Fig. **2**). Accordingly, the main point in this flowchart is the input data, then refining this data by multi-filtration steps, including removing the duplicated items and pseudogenes, and reaching multiple outputs which are based on the evinced-based clues of STRING-MODEL and need future functional validations. Of note, further investigations were performed based on the connected gene list from PPI, utilizing these refined data from the beginning to the end of the analysis.

## 3 RESULTS

### 3.1 Filtration Strategy and Primary Gene List

For the filtration process, we employed DrugBank, Ozempic (Semaglutide), GLP1R agonist, with four Enzymes (DPP4, MME, LPL, and AMY1A) related to GLP1 metabolism and one carrier in blood plasma albumen (ALB). Based on GeneCards score for Suicidal Ideation, the 20 highly scored genes were extracted and compared with 10 GARS genes (DRD1, DRD2, DRD3, DRD4, MAOA, COMT, DAT1 (SLC6A3), SLC6A4, OPRM1, and GABRA3). Remarkably, 4 genes of the GARS panel were common in the first 20 candidate genes for Suicide Ideation (SI). By combining the Ozempic target (GLP1R), related enzymes, and carrier genes to the 20 best-scored genes and 10 GARS genes, 31 genes remained for further analyses.

### 3.2 Primary *In silico* Analyses

#### 3.2.1 Protein-Protein Interactions (PPIs)

**Fig. (3):**
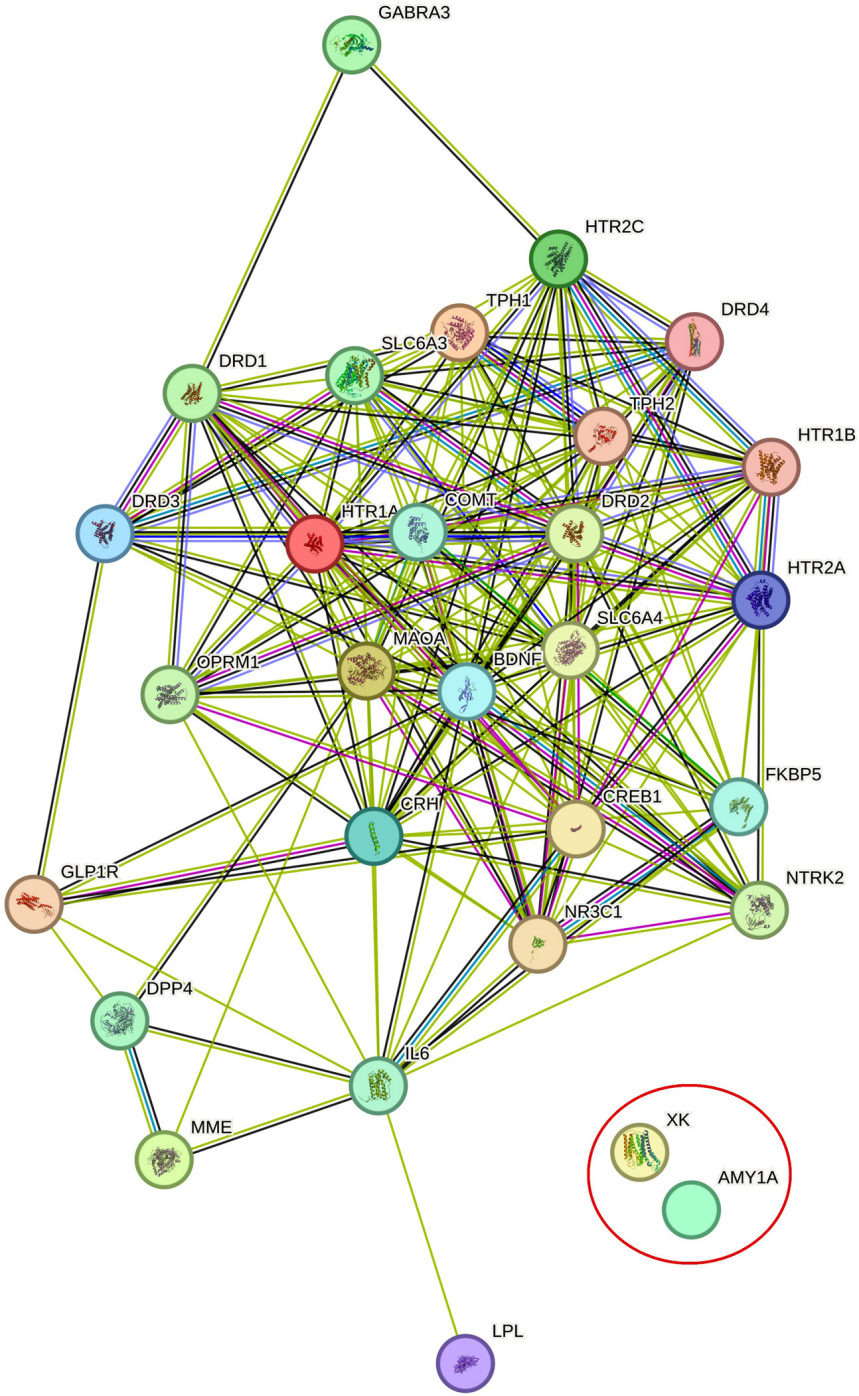
STRING-MODEL of PPIs for 31 candidate genes relating to Suicidal Ideation with GARS.

STRING-MODEL indicated a connected network of 29 genes out of 31 primary selected genes (PPI enrichment *p*-value < 1.0e-16). Thus, two unlinked proteins (AMY1A and XK) were eliminated. The connected genes are as follows: *GLP1R, SLC6A4, HTR2A, BDNF-AS, TPH2, LOC110806262 (SLC6A4 promoter), BDNF, TPH1, COMT, FKBP5, HTR1A, NR3C1, HTR1B, CRH, NTRK2, HTR2C, DRD2, IL6, MAOA, CREB1, DRD1, DRD3, DRD4, SLC6A3, OPRM1, GABRA3, DPP4, MME* and *LPL.* STRING-MODEL shows that *GLP1R* exhibits strong interactions with *DRD3, BDNF, CREB1, CRH, IL6,* and *DPP4* genes (Fig. **3**).

#### 3.2.2 Gene-regulatory Networks (GRNs)

Employing NetworkAnalyst (https://www.networkanalystca/NetworkAnalyst/), GRNs performed in Gene-miRNA Interactions (GMIs) and TF-miRNA revealed coregulation.

**Fig. (4):**
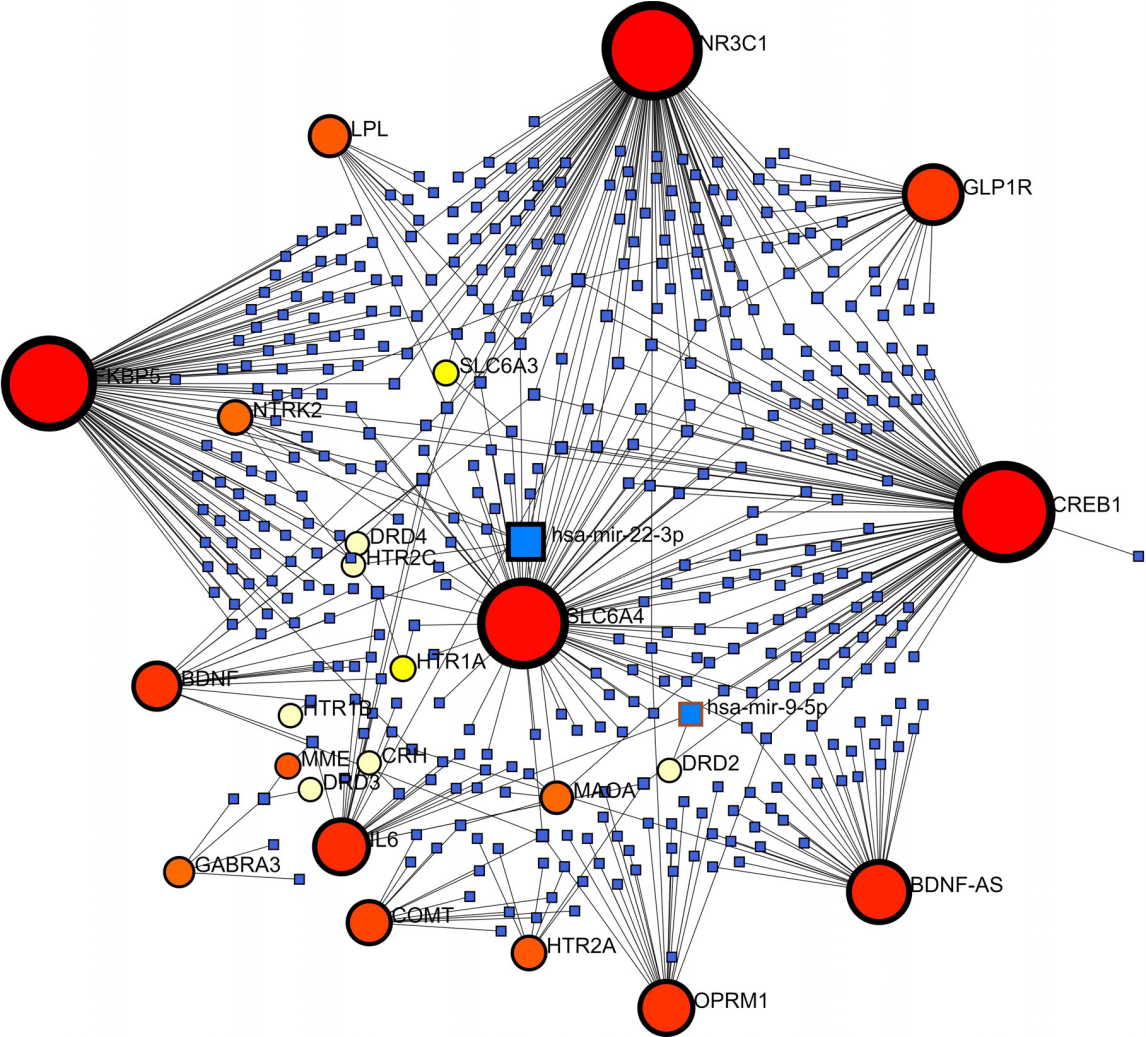
Force Atlas model of GMIs for 29 candidate genes including GARS genes representing CREB1 and hsa-miR-22-3p as the most interacting seed and miRNA, respectively.

We also validated GMIs data obtained from miRTarBase v.8.0. In a Force Atlas model, GMIs revealed that the CREB1 gene is the most interacted seed (betweenness degree = 107) and hsa-miR-22-3p has the most interactions (betweenness degree = 5) among other miRNAs. Interestingly, GLP1R interacted with CREB1 by hsa-miR-297 and hsa-miR-567. Moreover, hsa-miR-204-5p has links with GLP1R, CREB1, BDNF, NTRK2, and NR3C1 (Fig. **4**).

For TF-miRNA coregulatory networks, the literature curated regulatory interaction information that was collected from the RegNetwork repository. In a Linear Bi/Tripartite model, the results illustrated in Fig. (**5**) show the major impact of CREB1 (1728 interactions), NR3C1 (612 interactions), NTRK2 (70 interactions), BDNF (67 interactions), IL-6 (59 interactions), and SLC6A4 (47 interactions) as the best-scored protein targets. Moreover, SP1 and CTCF, both with 9 interactions, and REST, with 8 interactions, are the most interacted Transcription Factors (TFs). Additionally, the most significant miRNAs in this network are hsa-miR-16, hsa-miR-22, and hsa-miR-195, all with 6 interactions. Interestingly, SP1 and SP3 are TFs that have relationships with GLP1R, and OPRM1 has connections with these two TFs.

### 3.3 Deep *In silico* Identification through Enrichment Analysis

Enrichment analysis provides deep *in silico* identification, which was successfully performed on the candidate SI gene list suggested in this study. Enrichment analysis was categorized into three layers including Pathway Analysis, Gene Ontology (GO), and Disease/Drug Analysis. Pathway Analysis utilized Reactome, KEGG, and Panther databases. GO used GO Biological Process, GO Cellular Component, and GO Molecular Function; and Disease/Drug Analysis employed DisGENET, GeDiPNet 2023, and JENSEN DISEASE.

#### 3.3.1 Pathway Analysis

According to the Reactome 2022, following Amine Ligand-Binding Receptors R-HSA-375280 (*p*-value = 5.050e-16; adjusted *p*-value = 8.635e-14; OR = 237.37), Dopamine Receptors R-HSA-390651 (*p*-value =1.780e-11; adjusted *p*-value= 1.096e-9; OR = 3195.20) was the most significant pathway based on the 29 candidate genes of SI (Table **2**). Additionally, in KEGG 2021 HUMAN database classification, after Neuroactive ligand-receptor interaction (*p*-value = 4.347e-16; adjusted *p*-value = 4.869e-14; OR = 48.66) and Serotonergic synapse (*p*-value = 3.139e-12; adjusted *p*-value = 1.758e-10; OR = 72.08), the Dopaminergic synapse was in third place with a *p*-value of 1.110e-11, an adjusted *p*-value of 4.143e-10, and an OR of 60.97; thus, the significance of Dopaminergic synapse was not very different from the significance of Serotonergic synapses (adjusted *p*-value = 1.758e-10) (Table **3**). Finally, PANTHER listed the Dopamine receptor-mediated signaling pathway *Homo sapiens* P05912 as the most significant pathway (*p*-value = 1.040e-10; adjusted *p*-value = 1.768e-9; and OR = 113) for the gene list (Table **4**). Altogether, Pathway Analysis provided strong evidence for dopamine signaling in the SI phenotype.

#### 3.3.2 GO Analyses

Deep *in silico*/enrichment analyses based on GO Biological Process showed Dopaminergic Signaling (GO:0042417, *p*-value of 1.432e-13, adjusted *p*-value of 7.174e-11, and OR of 400.49) as the most associated with the SI phenotype (Table **5**). Analysis of GO Cellular Component 2023 indicated that Neuron Projection (GO:0043005, *p*-value of 7.589e-15, adjusted *p*-value of 2.656e-13, and OR of 33.39) is closely related to the SI phenotype (Table **6**). Moreover, Serotonin G Protein-Coupled Receptor Activity (GO:0004993) is the top-list of GO Molecular Function 2023 assessment (*p*-value = 8.920e-11; adjusted *p*-value = 6.779e-9; and OR = 259.83) (Table **7**). These findings support the idea that RDS and SI may share common mechanisms.

#### 3.3.3 Disease Drugs Interaction Analysis (DDA)

DDA was conducted by three databases: DisGeNET, GeDiPNet 2023, and JENSEN DISEASE, and their results are summarized in Table **8**. According to these results, the most significant disease phenotype was Major depressive disorder defined by Jensen DISEASE (*p*-value = 4.01E-40; adjusted *p-*value = 6.54e-38; and OR = 630.51) followed by Substance abuse (*p-*value = 2.20E-35; adjusted *p*-value = 1.80e-33; and OR = 681.54) and Alcohol dependence (*p*-value = 4.46E-35; adjusted *p*-value= 2.42e-33; and OR= 382.83). Noteworthy, the best models of DisGeNET and GeDiPNet 2023 were Mental depression (*p*-value = 3.72E-35; adjusted *p*-value = 9.12e-32; and OR = 220.69) and Mood disorder (*p*-value = 1.09E-31; adjusted *p*-value = 3.69E-29; and OR = 163.8), respectively (Table **8**).

To get a better view, Scatter plots, Manhattan plots, and Volcano plots for the Jensen DISEASE and DisGeNET are illustrated in Figs. (**6**, **7**, and **8**). The scatterplot was organized so that similar gene sets were clustered together. Larger, black-outlined points indicate significantly enriched terms. In the Manhattan plot, each line on the x-axis denoted a single gene set from the selected library, while the y-axis measured the –log_10_(*p*‐value) for each gene set. The volcano plot shows the significance of each gene set from the designated library *versus* its odds ratio. Each point displays a single Geneset; the x-axis measures the odds ratio (0, inf) calculated for the gene set, while the y-axis yields the -log(*p*-value) of the gene set. Larger blue points represent significant terms (*p*-value < 0.05); smaller gray points indicate non-significant terms. Also, the darker the blue color of a point, the more important it is.

## 4 DISCUSSION

In summary, by employing comprehensive *in silico* analysis, this study revealed genetic associations and close interaction between GLP1 signaling and specifically GLP1 receptor with 29 refined genes through STRING-MODEL, especially with *DRD3*, *BDNF*, *CREB1*, *CRH*, *IL6*, and *DPP4* genes. GMIs revealed that the *CREB1* gene is the most associated seed, and hsa-miR-22-3p has the most interactions among other miRNAs. Interestingly, GLP1R was associated with CREB1 through hsa-miR-297 and hsa-miR-567. Moreover, hsa-miR-204-5p has connections with GLP1R, CREB1, BDNF, NTRK2, and NR3C1. TF-miRNA coregulatory networks showed the major impact of CREB1, NR3C1, NTRK2, BDNF, IL-6, and SLC6A4 as the best-scored protein targets. Furthermore, SP1 and CTCF were the most interconnected TFs. Additionally, hsa-miR-16, hsa-miR-22, and hsa-miR-195 were found to be the most significant associated miRNAs in this network. Curiously, SP1 and SP3 were linked with GLP1R and OPRM1. According to the Pathways analyses, Dopamine Receptors R-HSA-390651, Dopaminergic synapse, and Dopamine receptor-mediated signaling pathway *Homo sapiens* P05912 were found as the top associate candidates. GO analyses showed Dopamine Metabolic Process (GO:0042417), Neuron Projection (GO:0043005), and G Protein-Coupled Serotonin Receptor Activity (GO:0004993) as the most significant processes. The most significant associated disease-causing phenotype among all three databases was major depressive disorder. Substance abuse and alcohol dependence were the second and third. Noteworthy, the best models of DisGeNET and GeDiPNet 2023 were both mental depression and mood disorders.

As mentioned earlier, a comprehensive gene list containing 29 genes was refined during a strategy of filtering the SI construct. These genes are included in the filtration according to the pharmacological and ligand-target interactions of Ozempic as the agonist of GLP1R and, three other enzymes and one plasma blood carrier. Additionally, we found the top 20 best-scored genes in GeneCards for “Suicide Ideation” as a keyword, including the 10 genes of GARS. Multiple analyses of these genes, including PPIs, GMIs, and TF-miR as a coregulatory network, revealed interesting results. Specifically, a deep *in silico* study of Enrichment Analysis was successfully carried out for this gene list. Pathway Analysis, GO analysis, and Disease Drug Analysis (DDA) all indicated that depressive phenotypes and dopamine regulation are the most significant associated factors playing remarkable roles in SI. Importantly, we caution against GLP1 agonist administration, especially in patients with known hypodopaminergia, rather than its general approval for long-term addiction treatment. However, our results were based on strong evidence and clinical reports in previous studies. Further clinical confirmation is required, and we acknowledge that the connection between neural transmission changes and SI remains speculative and multifactorial.

The majority of studies to date have indeed reported the benefits of Ozempic consumption. However, our study from a genetic standpoint indicated inconsistent findings. It is not possible to compare our studies with all of the reports due to some glaring reasons; this is not a review article, and we introduced a new perspective that requires further validation and confirmation. The FDA reported that they did not find an association between the use of the GLP-1 variant and suicidal thoughts or actions. However, they could not definitively rule out that a small risk may exist.

Despite these initial findings, we urge caution concerning attenuation rather than the chronic promotion of enhanced dopamine signaling, potentially leading to hypodopaminergic. Most recently, Wang *et al*., in a real-world cohort relying on electronic records, compared GLP1 agonistic therapy like semaglutide with other non-GLP1 agonist weight loss agents for depression and SI and could not support a relationship of induction of depression and SI for semaglutide [79]. While we are not refuting this important work by Wang *et al*. [79], albeit the issue whereby electronic records of patients may yield omissions related to certain diagnostic criteria (*e.g*., SI and even suicide attempts), we cannot ignore media reports such as the April 17^th^ from the NY Post suggesting that Ozempic appears to be changing a growing number of patients claiming that the GLP1 agonistic medications have caused anxiety, and SI even as they shed the pounds. It is also well-known that obese individuals also have a reduced dopaminergic function either genetically [24] or epigenetically [25], which only further complicates the issue at hand. However, it is indeed noteworthy that while we are in no position to assess the accuracy of the data collected from archival EMRs used in the Wang *et al*. study [79], there exists a plethora of literature reports (PubMed; Assessed 4-20-2024; 51,395 listings using EMR as a word search) that argue and or question the validity of utilizing EMR for assessing clinical outcomes and important questions [26-30]. Specifically, the secondary use of clinical data for research purposes is not without limitations. In accordance with the Preferred Reporting Items for Systematic Reviews and Meta-Analyses guidelines, Edmondson & Reimer [31] performed a systematic review to identify current issues related to the secondary use of electronic medical record data *via* MEDLINE and CINAHL databases. All articles published until June 2018 were included. Sixty articles remained after title and abstract review, and four domains of potential limitations were identified: (1) data quality issues, present in 91.7% of the articles reviewed; (2) data preprocessing challenges (53.3%); (3) privacy concerns (18.3%); and (4) potential for limited generalizability (21.7%).

Diabetes and insulin resistance are associated with altered brain imaging, depression, and increased rates of age-related cognitive impairment. Importantly, it has been demonstrated that mice with a brain-specific knockout of the insulin receptor (Neuronal Insulin Receptor Knockout (NIRKO) mice) exhibit brain mitochondrial dysfunction with reduced mitochondrial oxidative activity, increased levels of reactive oxygen species, and increased levels of lipid and protein oxidation in the striatum and nucleus accumbens [81, 82]; NIRKO mice also exhibit increased levels of monoamine oxidase A and B (MAO A and B) leading to increased dopamine turnover in these areas. This finding may exacerbate people with a genetic predisposition for schizophrenia, whereby reduced dopamine availability in the mitochondria could affect the clinical presence of schizophrenia and even depression [83, 84]. Studies in cultured neurons and glial cells indicate that these changes in MAO A and B are a direct consequence of loss of insulin signaling [85, 86]. As a result, NIRKO mice develop age-related anxiety and depressive-like behaviors that can be reversed by treatment with MAO inhibitors, as well as the tricyclic antidepressant imipramine, which inhibits MAO activity and reduces oxidative stress [87]. Also, insulin resistance in the brain induces mitochondrial and dopaminergic dysfunction, leading to anxiety and depressive-like behaviors, demonstrating a potential molecular link between central insulin resistance and behavioral disorders [88]. Another limitation arises from the controversy of various clinical reports related to this important topic. Interestingly, following careful consideration of the literature for investigating any match results with ours, we found a recent animal study by Sadek *et al*. conducted on the plausible impact of semaglutide on experimental autoimmune encephalomyelitis (EAE)-induced Multiple sclerosis in mice. They found semaglutide triggers the PI3K/Akt axis, which, in turn, inhibits the activity of GSK-3β. The inhibited GSK3β activity then decreases demyelination and causes remyelination *via* CREB/BDNF; moreover, it increases Nrf2 and SOD levels leading to protection in mice from EAE-induced oxidative stress [89]. A recently published report by Schoretsanitis *et al*. utilized the WHO database and found an association of Ozempic with suicidal ideation, which confirms our results [90].

## 5 LIMITATIONS

The current study has some limitations. For example, there is a lack of functional and molecular validation related to the link between pharmacological GLPR1 activation, depressive behavior, and SI. We utilized well-known sources with high reliability in our *in silico* and systems biology analyses, which might be augmented with additional sources or new sources in similar future studies. Another limitation *in silico* analysis is considered reporting every detail in future studies and neglecting the priorities (we basically reported the priorities/top-scored results). Additionally, transcriptomics and GWAS studies regarding Ozempic or other GLP1R agonists are limited; thus, our transcriptomic-associated results (potential miRNAs and Transcription Factors) remarkably need further validation on clinical samples. Our finding in the PPI network for BDNF should be further investigated in future genetic studies [91], as well as adverse effects, especially in terms of depression and RDS behaviors [92, 93].

## CONCLUSION

Despite the FDA's recent remarks that SI associated with GLP1R agonism is not a concern, these findings obviously show the negative impact of Ozempic as an agonist for GLP1 and its receptor (GLP1R). When Ozempic reaches the blood, it will begin to induce a number of reactions starting from GLP1 and its receptor. Based on our silico predictions, the binding of Ozempic to GLP1 receptor will activate several pathways, which may lead to the dysregulation of the dopaminergic signaling and, as a consequence, exacerbation of depressive behaviors, which are one of the main endophenotypes of RDS. Notably, the associations found in the *in silico* analysis require further empirical validation through clinical studies.

## Figures and Tables

**Fig. (5):**
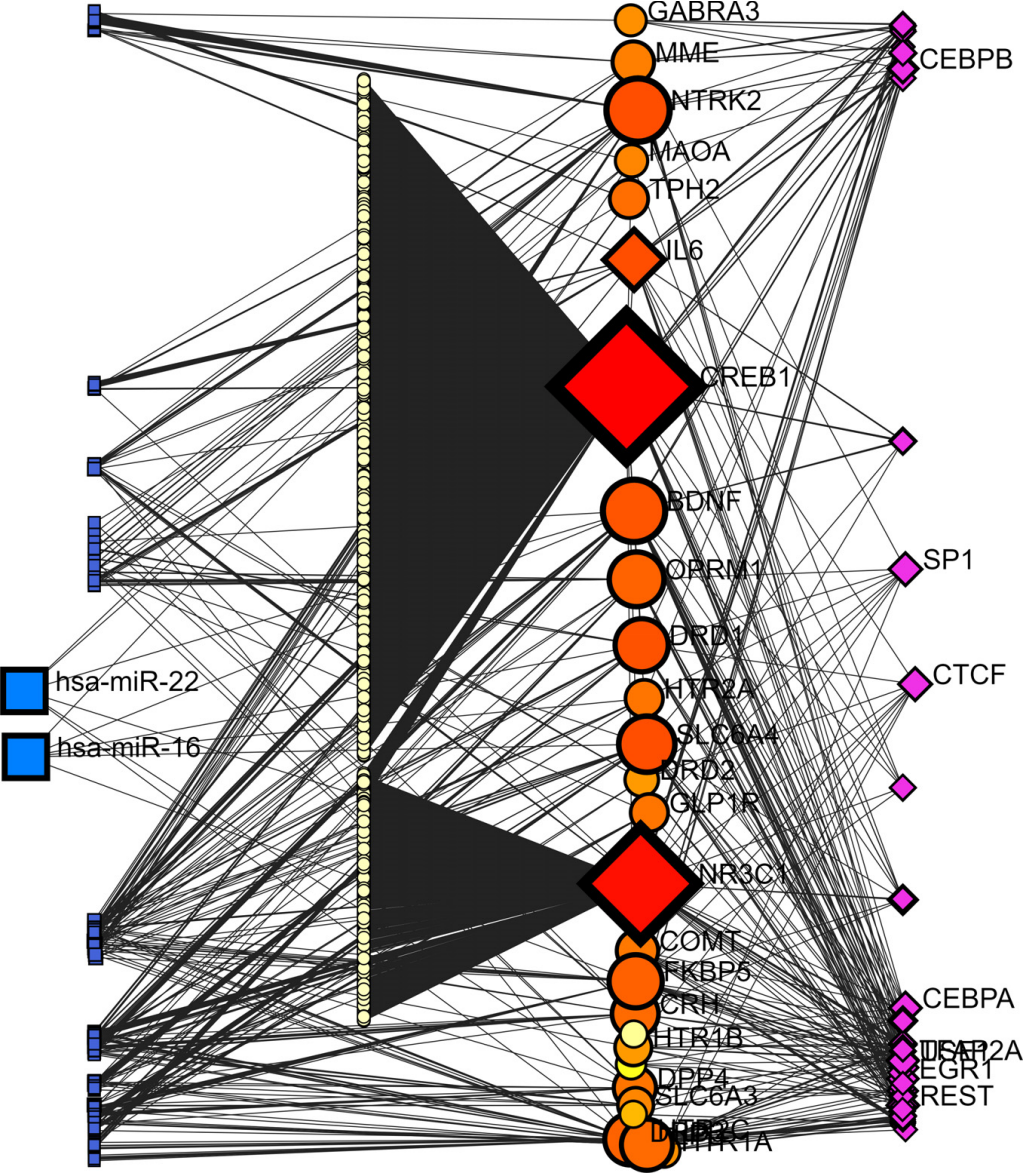
The Linear Bi/Tripartite model is built on the TF-miRNA coregulatory network of 29 SI candidate genes. According to this figure, CREB1, SP1, and miR-16 and -22 are the most important protein targets, TF, and miRNAs, respectively.

**Fig. (6):**
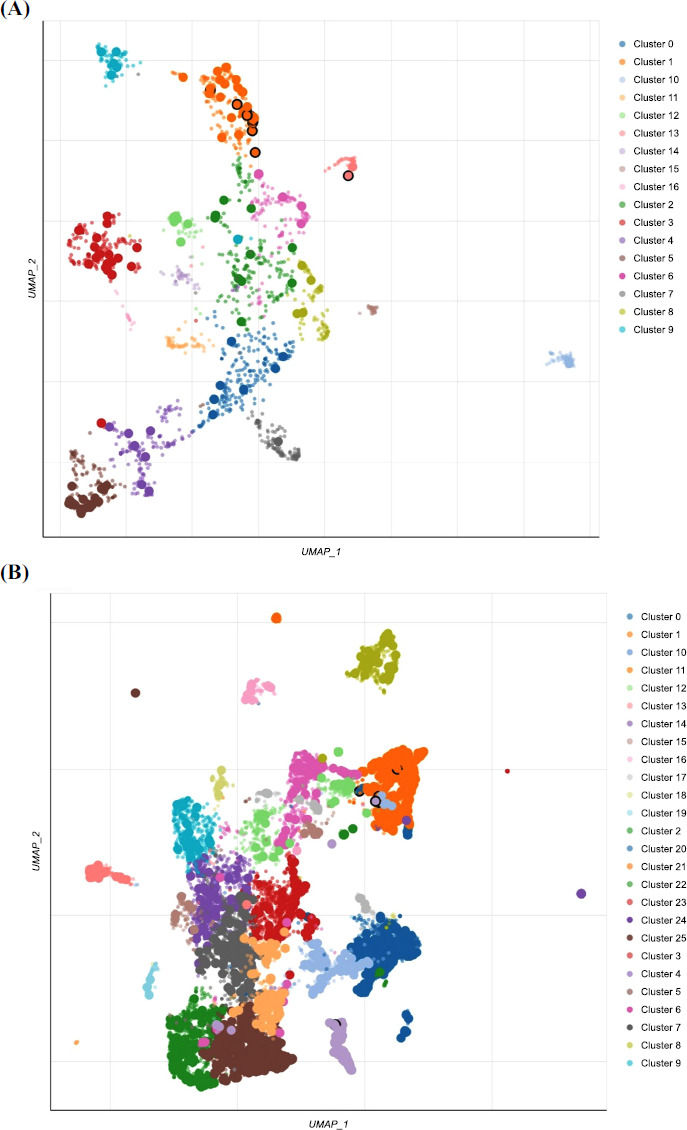
Scatterplot of all terms in Jensen Disease database (**A**) and DisGeNET database (**B**). Each point signifies a term in the library. Term frequency-inverse document frequency (TF-IDF) values were computed for the gene set corresponding to each term, and UMAP was utilized to display the resulting values. The terms are plotted based on the first two UMAP dimensions. Generally, terms with more similar gene sets are positioned closer together. Terms are colored by automatically identified clusters computed with the Leiden algorithm applied to the TF-IDF values. The darker and larger the point, the more significantly enriched the term. Hovering over points will display the term, the *p*-value from the enrichment calculation, and the automatically assigned cluster.

**Fig. (7):**
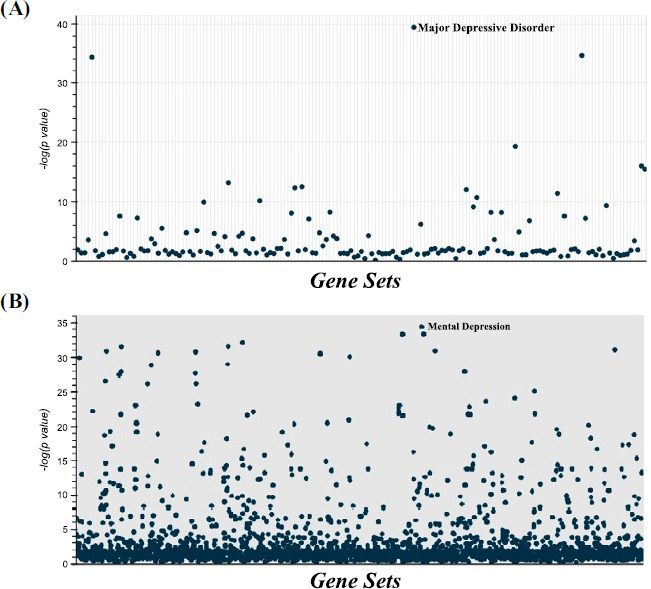
Manhattan plot of terms from Jensen Disease database (**A**) and DisGeNET database (**B**) for SI candidate gene list indicating Depression as the most top hub. Each point represents a single term along the x-axis. The y-values represent the -log10(*p*-value) corresponding to the enrichment of the input gene set for the term gene set.

**Fig. (8):**
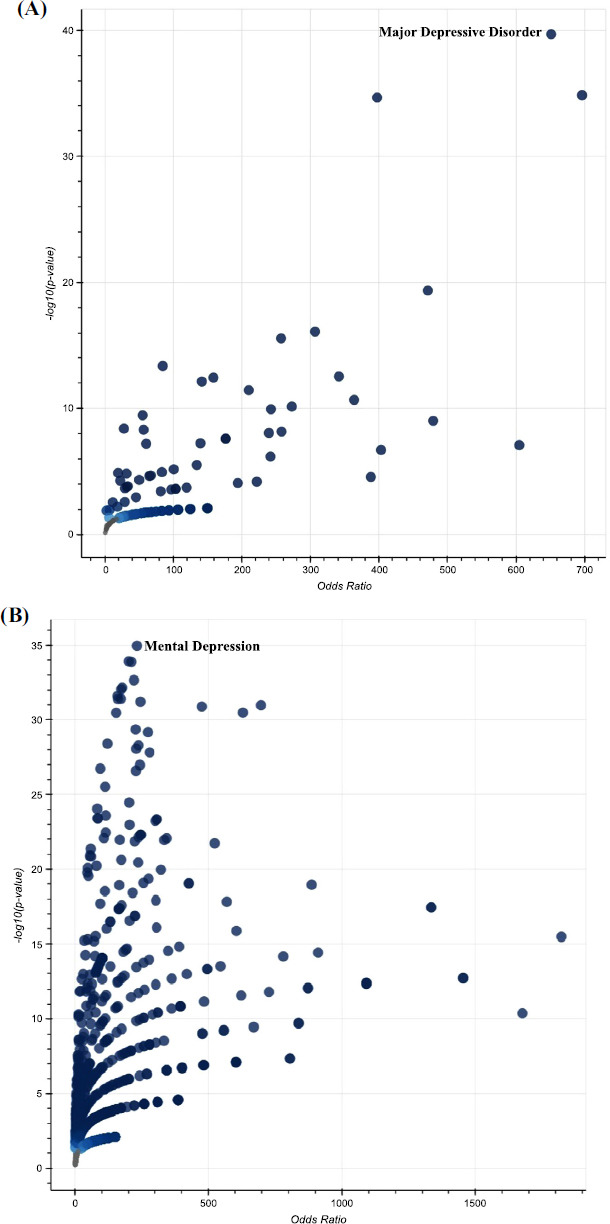
Volcano plot of terms from Jensen Disease database (**A**) and DisGeNET database (**B**) for SI candidate gene list showing depression phenotype in the top. Each point shows a single term, plotted by the corresponding odds ratio (x-position) and -log10(*p*-value) (y-position) from the enrichment results of the input query gene set. The larger and darker-colored the point, the more significantly enriched the input gene set is for the term.

**Table 1:** Contents, validations, and references of databases and systems biology approaches applied in this paper.

**Level**	**Database**	**Site**	**Software (Version)**	**References**
PPIs	String-Model	https://string-db.org/	STRING (12.0)	[17]
GRNs	GMIs (miRTarBase)	https://mirtarbase.cuhk.edu.cn/~miRTarBase/miRTarBase_2022/php/index.php	NetworkAnalyst (3.0)	[18]
	TF-coregulatory Interactions	http://www.regnetworkweb.org/	NetworkAnalyst (3.0)	[19]
EA	Pathway Analysis	https://maayanlab.cloud/Enrichr/	Enrichr	[20]
	GO	https://maayanlab.cloud/Enrichr/	Enrichr	[21]
	DDA	https://maayanlab.cloud/Enrichr/	Enrichr	[22]

**Abbreviations:** PPIs: Protein-Protein Interactions; GRNs: Gene Regulatory Networks; GMIs: Gene-miRNA Interactions; EA: Enrichment Analysis; GO: Gene Ontology; and DDA: Diseases Drugs Assessment.

**Table 2:** Reactome 2022 results for pathway analysis of 21 candidate genes list related to suicide ideation.

**Index**	**Name**	***P*-value**	**q-value**	**OR**
1	Amine Ligand-Binding Receptors R-HSA-375280	5.050e-16	8.635e-14	237.37
2	**Dopamine Receptors R-HSA-390651**	**1.780e-11**	**1.096e-9**	**3195.20**
3	GPCR Ligand Binding R-HSA-500792	1.923e-11	1.096e-9	26.69
4	Signaling By GPCR R-HSA-372790	7.703e-11	3.293e-9	20.12
5	Class A/1 (Rhodopsin-like Receptors) R-HSA-373076	5.619e-10	1.922e-8	27.81
6	Neurotransmitter Clearance R-HSA-112311	7.437e-10	2.120e-8	532.40
7	Serotonin Receptors R-HSA-390666	1.168e-9	2.852e-8	456.32
8	Signal Transduction R-HSA-162582	3.963e-9	8.470e-8	10.14
9	GPCR Downstream Signaling R-HSA-388396	8.833e-9	1.678e-7	16.73
10	Transcriptional Regulation By MECP2 R-HSA-8986944	2.303e-8	3.938e-7	75.44

**Note**: OR refers to Odds ration and q-value is calculated based on adjusted *p*-value.

**Table 3:** KEGG HUMAN 2021 phenotypes resulted from 29 candidate genes.

**Index**	**Name**	***P*-value**	**q-value**	**OR**
1	Neuroactive ligand-receptor interaction	4.347e-16	4.869e-14	48.66
2	Serotonergic synapse	3.139e-12	1.758e-10	72.08
3	**Dopaminergic synapse**	**1.110e-11**	**4.143e-10**	**60.97**
4	Cocaine addiction	7.166e-11	2.006e-9	120.90
5	Alcoholism	1.747e-10	3.914e-9	42.36
6	cAMP signaling pathway	1.983e-8	3.702e-7	30.09
7	Amphetamine addiction	0.000002887	0.00004619	49.00
8	Gap junction	0.000007640	0.0001070	37.88
9	Tryptophan metabolism	0.00003029	0.0003769	58.97
10	Taste transduction	0.0002587	0.002897	27.65

**Note**: OR means Odds Ration and q-value stands for adjusted *p*-value.

**Table 4:** PANTHER pathways for candidate gene list of SI.

**Index**	**Name**	***P*-value**	**q-value**	**OR**
1	Dopamine receptor mediated signaling pathway *Homo sapiens* P05912	1.040e-10	1.768e-9	113.00
2	Heterotrimeric G-protein signaling pathway-Gi alpha and Gs alpha mediated pathway *Homo sapiens* P00026	2.453e-8	2.085e-7	42.80
3	Adrenaline and noradrenaline biosynthesis *Homo sapiens* P00001	0.000006170	0.00003496	104.63
4	5HT1 type receptor mediated signaling pathway *Homo sapiens* P04373	0.000009764	0.00004150	88.51
5	5HT2 type receptor mediated signaling pathway *Homo sapiens* P04374	0.00003990	0.0001356	53.47
6	Enkephalin release *Homo sapiens* P05913	0.0002724	0.0007718	98.55
7	Heterotrimeric G-protein signaling pathway-Gq alpha and Go alpha mediated pathway *Homo sapiens* P00027	0.0004783	0.001162	22.26
8	Nicotine pharmacodynamics pathway *Homo sapiens* P06587	0.0007496	0.001593	56.82
9	5-Hydroxytryptamine degradation *Homo sapiens* P04372	0.007230	0.01366	178.28
10	5HT3 type receptor mediated signaling pathway *Homo sapiens* P04375	0.02296	0.03548	47.51

**Note**: q-value is computated based on adjusted *p*-value and OR refers to Odds Ration.

**Table 5:** List of genes associated with suicide ideation (based on GO biological process database, 2023).

**Index**	**Name**	***P*-value**	**q-value**	**OR**
1	Dopamine Metabolic Process (GO:0042417)	1.432e-13	7.174e-11	400.49
2	G Protein-Coupled Receptor (GPCR) Signaling Pathway, (GO:0007187)	5.893e-13	1.476e-10	147.46
3	Catecholamine Metabolic Process (GO:0006584)	5.687e-12	9.497e-10	519.87
4	Adenylate Cyclase-Inhibiting GPCR Signaling Pathway (GO:0007193)	1.040e-10	1.303e-8	113.00
5	GPCR Serotonin Signaling Pathway (GO:0098664)	4.467e-10	4.476e-8	638.91
6	Phospholipase C-mediated Signaling Pathway (GO:0007200)	1.870e-9	1.562e-7	67.40
7	Chemical Synaptic Transmission (GO:0007268)	3.642e-9	2.607e-7	28.33
8	Response To Organonitrogen Compound (GO:0010243)	6.552e-9	4.103e-7	98.85
9	Response to Ethanol (GO:0045471)	1.075e-8	5.985e-7	228.08
10	Behavioral Fear Response (GO:0001662)	2.735e-8	0.000001246	1152.06

**Abbreviations**: GO: Gene Ontology; q-value: adjusted *p*-value; and OR: Odds Ratio.

**Table 6:** Results of GO cellular component 2023 for SI candidate gene list.

**Index**	**Name**	***P*-value**	**q-value**	**OR**
1	Neuron Projection (GO:0043005)	7.589e-15	2.656e-13	33.39
2	Dendrite (GO:0030425)	6.176e-14	1.081e-12	46.51
3	Axon (GO:0030424)	4.206e-7	0.000004906	25.92
4	Non-Motile Cilium (GO:0097730)	0.0008611	0.007535	52.76
5	Ciliary Membrane (GO:0060170)	0.001382	0.009672	41.02
6	Membrane Raft (GO:0045121)	0.001843	0.01075	13.77
7	Flotillin Complex (GO:0016600)	0.008670	0.04335	142.61
8	Cell Projection Membrane (GO:0031253)	0.01011	0.04421	14.29
9	Focal Adhesion (GO:0005925)	0.01810	0.06685	5.89
10	Cell-Substrate Junction (GO:0030055)	0.01910	0.06685	5.76

**Note**: GO and OR stand for Gene ontology and Odds Ratio, respectively.

**Table 7:** The categorized list of GO Molecular Function 2023 for candidate gene list involving in SI.

**Index**	**Name**	***P*-value**	**q-value**	**OR**
1	GPCR Serotonin Activity (GO:0004993)	8.920e-11	6.779e-9	259.83
2	Serotonin Receptor Activity (GO:0099589)	2.869e-10	1.084e-8	197.92
3	G Protein-Coupled Amine Receptor Activity (GO:0008227)	4.278e-10	1.084e-8	180.69
4	Monoamine Transmembrane Transporter Activity (GO:0008504)	0.0001108	0.002104	164.30
5	Sodium:Chloride Symporter Activity (GO:0015378)	0.0001568	0.002383	134.41
6	Postsynaptic Neurotransmitter Receptor Activity (GO:0098960)	0.0003419	0.004330	86.95
7	GPCR Activity (GO:0004930)	0.0004427	0.004806	12.83
8	Receptor Ligand Activity (GO:0048018)	0.001101	0.01046	9.98
9	Exopeptidase Activity (GO:0008238)	0.002574	0.02173	29.51
10	Growth Factor Activity (GO:0008083)	0.006727	0.04118	17.75

**Abbreviations**: GPCR: G-protein Coupled Receptor; GO: Gene Ontology; OR: Odds Ratio.

**Table 8:** Candidate genes of SI phenotype (29 genes) extracted from combined results from DisGeNET, GeDiPNET, and JENSEN DISEASE databases.

**Index**	**Name**	**Database**	**P-value**	**q-value**	**OR**
1	Major Depressive Disorder	JENSEN DISEASE	4.01E-40	6.54E-38	630.51
2	Substance Abuse	JENSEN DISEASE	2.20E-35	1.80E-33	681.54
3	Alcohol Dependence	JENSEN DISEASE	4.46E-35	2.42E-33	382.83
4	Mental Depression	DisGeNET	3.72E-35	9.12E-32	220.69
5	Mental Disorders	DisGeNET	3.94E-34	3.32E-31	201.31
6	Major Depressive Disorder	DisGeNET	4.06E-34	3.32E-31	191.23
7	Drug Abuse	DisGeNET	5.88E-33	3.61E-30	210.12
8	Depressive Disorder	DisGeNET	2.30E-32	1.05E-29	168.08
9	Anxiety Disorders	DisGeNET	2.58E-32	1.05E-29	164.68
10	Unipolar Depression	DisGeNET	7.76E-32	2.72E-29	151.14
11	Mood Disorders	DisGeNET	1.09E-31	3.25E-29	163.8
12	Alcoholic Intoxication, Chronic	DisGeNET	1.19E-31	3.25E-29	152.99
13	Cocaine Dependence	DisGeNET	1.51E-31	3.69E-29	233.47
14	Mood Disorder	GeDiPNet	1.65E-31	6.38E-29	176.01
15	Bipolar Disorder	GeDiPNet	7.60E-27	1.47E-24	89.84
16	Mental Depression	GeDiPNet	4.79E-26	6.18E-24	83.8
17	Personality Disorder	JENSEN DISEASE	4.82E-20	1.97E-18	472.55
18	Schizophrenia	GeDiPNet	3.34E-18	3.23E-16	33.53
19	Obsessive-compulsive Disorder	JENSEN DISEASE	9.18E-17	2.99E-15	303.94
20	Post-traumatic Stress Disorder	JENSEN DISEASE	3.22E-16	8.74E-15	253.22
21	Jacksonian Seizure	GeDiPNet	1.37E-14	1.06E-12	97.23
22	Dementia	JENSEN DISEASE	6.23E-14	1.45E-12	81.25
23	Clonic Seizures	GeDiPNet	5.33E-14	3.44E-12	82.76
24	Heroin Dependence	JENSEN DISEASE	2.86E-13	5.82E-12	347.06
25	Gilles de la Tourette Syndrome	JENSEN DISEASE	4.35E-13	7.88E-12	154.67
26	Hypotonic Seizures	GeDiPNet	2.42E-13	1.34E-11	69.22
27	Nicotine Dependence	JENSEN DISEASE	9.07E-13	1.48E-11	137.82
28	Anhedonia	GeDiPNet	7.07E-13	3.42E-11	289.17
29	Manic Disorder	GeDiPNet	1.66E-11	7.15E-10	87.94
30	Melancholia	GeDiPNet	2.32E-11	8.99E-10	148.59

**Note**: q-value and OR refer to adjusted *p*-value and Odds Ratio, respectively.

## Data Availability

The data and supportive information are available within the article.

## LIST OF ABBREVIATIONS

GLP1R: Glucagon-like peptide-1 Receptor
SUD: Substance Use Disorder
AUD: Alcohol Use Disorder
SI: Suicidal Ideation
RDS: Reward Deficiency Syndrome
VTA: Ventral Tegmental Area
NAc: Nucleus Accumbens
GLP-1: Glucagon-like Peptide 1
ASCVD: Atherosclerotic Cardiovascular Disease
fMRI: Functional MRI
GO: Gene Ontology
SI: Suicide Ideation
PPIs: Protein-protein Interactions
GRNs: Gene-regulatory Networks
DDA: Disease Drug Analysis
