# Supplementary material for: In Silico Pharmacogenomic Assessment of Glucagon-like Peptide-1 (GLP1) Agonists and the Genetic Addiction Risk Score (GARS) Related Pathways: Implications for Suicidal Ideation and Substance Use Disorder
Source: Curr Neuropharmacol. 2025 Jan 24;23(8):974–95. doi: 10.2174/011570159X349579241231080602 (PMC12174944; doi:10.2174/011570159X349579241231080602)
Supplement: Supplementary file 1 [file CN-23-8-974_SD1.pdf]

Supplementary Material

In Silico Pharmacogenomic Assessment of Glucagon-like Peptide-1 (GLP1) Agonists and the Genetic Addiction Risk Score (GARS) Related Pathways: Implications for Suicidal Ideation and Substance Use Disorder

Alireza Sharafshah[1], Kai-Uwe Lewandrowski[2,3], Mark S. Gold[4], Brian Fuehrlein[5], John Wesson Ashford[6], Panayotis K. Thanos[7,17], Gene Jack Wang[8], Colin Hanna[7], Jean Lud Cadet[9], Eliot L. Gardner[10], Jag H. Khalsa[11,12], Eric R. Braverman[13], David Baron[14], Igor Elman[15,17,*], Catherine A. Dennen[16], Abdalla Bowirrat[17], Albert Pinhasov[17], Edward J. Modestino[18], Paul R. Carney[19], Rene Cortese[20], Rossano Kepler Alvim Fiorelli[21], Sergio Schmidt[22], Aryeh R. Pollack[13], Rajendra D. Badgaiyan[13,23], Kenneth Blum[3,13,14,17, 20,24,25,*]

[1]Cellular and Molecular Research Center, School of Medicine, Guilan University of Medical Sciences, Rasht, Iran; [2]Department of Orthopaedics, Fundación Universitaria Sanitas Bogotá D.C. Colombia; [3]Division Personalized Pain Research and Education, Center for Advanced Spine Care of Southern Arizona, Tucson, AZ., 85712, USA; [4]Department of Psychiatry, Washington University School of Medicine, St. Louis, MO., 63110, USA; [5]Department of Psychiatry, Yale University School of Medicine, New Haven CT., 06511, USA; [6]Department of Psychiatry & Behavioral Sciences, Stanford University, Palo Alto, CA Director, War Related Illness & Injury Study Center, VA Palo Alto Health Care System, Palo Alto, CA, 94305, USA; [7]Behavioral Neuropharmacology and Neuroimaging Laboratory on Addictions, Clinical Research Institute on Addictions, Department of Pharmacology and Toxicology, Jacobs School of Medicine and Biosciences, State University of New York at Buffalo, Buffalo, NY., 14260, USA; [8]Laboratory of Neuroimaging, National Institute of Alcohol Abuse & Alcoholism, Bethesda, MD, 20892, United States; [9]Molecular Neuropsychiatry Research Branch, NIH National Institute on Drug Abuse, Bethesda, MD., 20892, USA; [10]Neuropsychopharmacology Section, Intramural Research Program, National Institute on Drug Abuse, National Institutes of Health, Baltimore, MD., 20892, USA; [11]Division of Therapeutics and Medical Consequences, Medical Consequences of Drug Abuse and Infections Branch, National Institute on Drug Abuse, NIH, Special Volunteer, Industrial Drive, Gaithersburg, MD., 20892, The USA; [12]Department of Microbiology, Immunology, and Tropical Medicine, The George Washington University School of Medicine, Washington, NWDC., 20037, The USA; [13]The Kenneth Blum Behavioral & Neurogenetic Institute, LLC., Austin, TX., 78701, USA; [14]Center for Sports, Exercise, Mental Health, Western University Health Sciences, Lebanon, OR., 91766, USA; [15]Cambridge Health Alliance, Harvard Medical School, Cambridge, MA., 02115, USA; [16]Department of Family Medicine, Jefferson Health Northeast, Philadelphia, PA., USA; [17]Department of Molecular Biology, Adelson School of Medicine, Ariel University, Ariel, Israel; [18]Brain & Behavior Laboratory, Department of Psychology, Curry College, Milton, MA., 02186, USA; [19]Departments of Pediatrics and Neurology, University of Missouri School of Medicine, Columbia, 65212, Missouri, USA; [20]Departments of Pediatrics and Obstetrics, Gynecology and Women's Health. School of Medicine. University of Missouri, Columbia, MO, 65212, USA; [21]Department of General and Specialized Surgery, Gaffrée e Guinle University Hospital, Federal University of the State of Rio de Janeiro (UNIRIO), Rio de Janeiro, Brazil; [22]Post-Graduate Program in Neurology, Federal University of the State of Rio de Janeiro, Rio de Janeiro, Brazil; [23]Department of Psychiatry, Texas Tech University Health Sciences, School of Medicine, Midland, TX., 79430, USA; [24]Department of Psychiatry, Wright State University Boonshoft School of Medicine and Dayton VA Medical Center, Dayton, OH., 45435, USA; [25]Department of Psychiatry, Human Integrated Services Unit, University of Vermont Center for Clinical & Translational Science, College of Medicine, Burlington, VT, 05401, USA

1875-6190/25 $65.00+.00 **© 2025 Bentham Science Publishers**

Supplementary Table 1. The rationale behind the including the GARS genes in the primary gene list of this study.

Gene	Depression Studies - PubMed	Reference An Example	GWAS
DRD1	73	Chen H, Chen J, Lan J. Acute manipulation of Drd1 neurons in the prefrontal cortex bidirectionally regulates anxiety and depression-like behaviors. Neurosci Lett. 2024 May 29;832:137805. doi: 10.1016/j.neulet.2024.137805. Epub 2024 May 4. PMID: 38705453.	NONE
DRD2	264	Chen H, Xiong XX, Jin SY, He XY, Li XW, Yang JM, Gao TM, Chen YH. Dopamine D2 receptors in pyramidal neurons in the medial prefrontal cortex regulate social behavior. Pharmacol Res. 2024 Jan;199:107042. doi: 10.1016/j.phrs.2023.107042. Epub 2023 Dec 22. PMID: 38142878.	16<br>Levey DF, Stein MB, Wendt FR, Pathak GA, Zhou H, Aslan M, Quaden R, Harrington KM, Nuñez YZ, Overstreet C, Radhakrishnan K, Sanacora G, McIntosh AM, Shi J, Shringarpure SS; 23andMe Research Team; Million Veteran Program; Concato J, Polimanti R, Gelernter J. Bi-ancestral depression GWAS in the Million Veteran Program and meta-analysis in >1.2 million individuals highlight new therapeutic directions. Nat Neurosci. 2021 Jul;24(7):954-963. doi: 10.1038/s41593-021-00860-2. Epub 2021 May 27. PMID: 34045744; PMCID: PMC8404304.
DRD3	97	Xu L, Zhang J, Yang H, Cao C, Fang R, Liu P, Luo S, Wang B, Zhang K, Wang L. Epistasis in neurotransmitter receptors linked to posttraumatic stress disorder and major depressive disorder comorbidity in traumatized Chinese. Front Psychiatry. 2024 Feb 29;15:1257911. doi: 10.3389/fpsyt.2024.1257911. PMID: 38487579; PMCID: PMC10937445.	1<br>Fitzgerald E, Arcego DM, Shen MJ, O'Toole N, Wen X, Nagy C, Mostafavi S, Craig K, Silveira PP, Rayan NA, Diorio J, Meaney MJ, Zhang TY. Sex and cell-specific gene expression in corticolimbic brain regions associated with psychiatric disorders revealed by bulk and single-nuclei RNA sequencing. EBioMedicine. 2023 Sep;95:104749. doi: 10.1016/j.ebiom.2023.104749. Epub 2023 Aug 5. PMID: 37549631; PMCID: PMC10432187.
DRD4	132	Nasir Hashmi A, Sabina Raja M, Taj R, Ahmed Dharejo R, Agha Z, Qamar R, Azam M. Association of 11 variants of the dopaminergic and cognitive pathways genes with major depression, schizophrenia and bipolar disorder in the Pakistani population. Int J Neurosci. 2023 Aug 29:1-13. doi: 10.1080/00207454.2023.2251661. Epub ahead of print. PMID: 37642370.	4<br>Lai JH, Zhu YS, Huo ZH, Sun RF, Yu B, Wang YP, Chai ZQ, Li SB. Association study of polymorphisms in the promoter region of DRD4 with schizophrenia, depression, and heroin addiction. Brain Res. 2010 Nov 4;1359:227-32. doi: 10.1016/j.brainres.2010.08.064. Epub 2010 Aug 27. PMID: 20801104.
DAT1	245	Bahi A, Dreyer JL. Dopamine transporter (DAT) knockdown in the nucleus accumbens improves anxiety- and depression-related behaviors in adult mice. Behav Brain Res. 2019 Feb 1;359:104-115. doi: 10.1016/j.bbr.2018.10.028. Epub 2018 Oct 24. PMID: 30367968.	8<br>Eyre HA, Eskin A, Nelson SF, St Cyr NM, Siddarth P, Baune BT, Lavretsky H. Genomic predictors of remission to antidepressant treatment in geriatric depression using genome-wide expression analyses: a pilot study. Int J Geriatr Psychiatry. 2016 May;31(5):510-7. doi: 10.1002/gps.4356. Epub 2015 Oct 15. PMID: 26471432; PMCID: PMC5567872.
5-HTT	1,697	López-Echeverri YP, Cardona-Londoño KJ, Garcia-Aguirre JF, Orrego-Cardozo M. Effects of serotonin transporter and receptor polymorphisms on depression. Rev Colomb Psiquiatr (Engl Ed). 2023 Apr-Jun;52(2):130-138. English, Spanish. doi: 10.1016/j.rcpeng.2021.07.003. Epub 2023 Jul 13. PMID: 37453823.	34<br>Garvert L, Kirchner K, Grabe HJ, Van der Auwera S. Genome-wide gene-gene interaction of the 5-HTTLPR promoter polymorphism emphasizes the important role of neuroplasticity in depression. Prog Neuropsychopharmacol Biol Psychiatry. 2022 Dec 20;119:110614. doi: 10.1016/j.pnpbp.2022.110614. Epub 2022 Aug 14. PMID: 35977647.
OPRM1	23	Swann G, Byck GR, Dick DM, Aliev F, Latendresse SJ, Riley B, Kertes D, Sun C, Salvatore JE, Bolland J, Mustanski B. Effect of OPRM1 and stressful life events on symptoms of major depression in African American adolescents. J Affect Disord. 2014 Jun;162:12-9. doi: 10.1016/j.jad.2014.03.020. Epub 2014 Mar 27. PMID: 24766998; PMCID: PMC4083691.	1<br>Zhou H, Rentsch CT, Cheng Z, Kember RL, Nunez YZ, Sherva RM, Tate JP, Dao C, Xu K, Polimanti R, Farrer LA, Justice AC, Kranzler HR, Gelernter J; Veterans Affairs Million Veteran Program. Association of OPRM1 Functional Coding Variant With Opioid Use Disorder: A Genome-Wide Association Study. JAMA Psychiatry. 2020 Oct 1;77(10):1072-1080. doi: 10.1001/jamapsychiatry.2020.1206. Erratum in: JAMA Psychiatry. 2021 Feb 1;78(2):224. doi: 10.1001/jamapsychiatry.2020.3485. PMID: 32492095; PMCID: PMC7270886.

Gene	Depression Studies - PubMed	Reference An Example	GWAS
MAO-A	290	Toledo-Lozano CG, López-Hernández LB, Suárez-Cuenca JA, Villalobos-Gallegos L, Jiménez-Hernández DA, Alcaraz-Estrada SL, Mondragón-Terán P, Joya-Laureano L, Coral-Vázquez RM, García S. Individual and Combined Effect of *MAO-A*/*MAO-B* Gene Variants and Adverse Childhood Experiences on the Severity of Major Depressive Disorder. Behav Sci (Basel). 2023 Sep 26;13(10):795. doi: 10.3390/bs13100795. PMID: 37887445; PMCID: PMC10603972.	6<br>Huang SY, Lin MT, Lin WW, Huang CC, Shy MJ, Lu RB. Association of monoamine oxidase A (MAOA) polymorphisms and clinical subgroups of major depressive disorders in the Han Chinese population. World J Biol Psychiatry. 2009;10(4 Pt 2):544-51. doi: 10.1080/15622970701816506. PMID: 19224413.
COMT	480	Sanabrais-Jiménez MA, Aguilar-García A, Hernández-Muñoz S, Sarmiento E, Ulloa RE, Jiménez-Anguiano A, Camarena B. Association study of Catechol-O-Methyltransferase (*COMT*) rs4680 Val158Met gene polymorphism and suicide attempt in Mexican adolescents with major depressive disorder. Nord J Psychiatry. 2022 Apr;76(3):202-206. doi: 10.1080/08039488.2021.1945682. Epub 2021 Aug 3. PMID: 34342556.	12<br>Maul S, Giegling I, Fabbri C, Corponi F, Serretti A, Rujescu D. Genetics of resilience: Implications from genome-wide association studies and candidate genes of the stress response system in posttraumatic stress disorder and depression. Am J Med Genet B Neuropsychiatr Genet. 2020 Mar;183(2):77-94. doi: 10.1002/ajmg.b.32763. Epub 2019 Oct 4. PMID: 31583809.
GABRB3	1	Bergen SE, Fanous AH, Walsh D, O'Neill FA, Kendler KS. Polymorphisms in SLC6A4, PAH, GABRB3, and MAOB and modification of psychotic disorder features. Schizophora Res. 2009 Apr;109(1-3):94-7. doi: 10.1016/j.schres.2009.02.009. Epub 2009 Mar 5. PMID: 19268543; PMCID: PMC2682723.	NONE
